# The injuries to the fourth and fifth tarsometatarsal joints: A review of the surgical management by internal fixation, arthrodesis and arthroplasty

**DOI:** 10.12669/pjms.292.2996

**Published:** 2013-04

**Authors:** Xiao Yu, Qing-jiang Pang, Guang-rong Yu

**Affiliations:** 1 Xiao Yu, Department of Orthopedics Centre, Ningbo No.2 Hospital, Ningbo 315010, Zhejiang, China.; 2 Qing-jiang Pang, Department of Orthopedics Centre, Ningbo No.2 Hospital, Ningbo 315010, Zhejiang, China.; 3 Guang-rong Yu, Department of Orthopedics, Tongji Hospital, Tongji University School of Medicine, Shanghai 20065, China.

**Keywords:** Tarsometatarsal joint, Open reduction and internal fixation, Arthroplasty, Arthrodesis

## Abstract

The surgical management to the injuries of the fourth and fifth tarsometatarsal (TMT) joints is controversial. We briefly review the anatomical characteristics to the injuries, the diagnosis, as well as the individualized treatment of the injuries of the fourth and fifth TMT joints by open reduction and internal fixation, TMT arthrodesis and arthroplasty. We conclude that open reduction and internal fixation is the recommended option for acute injuries, while arthrodesis can be used in cases of malunion of the fourth and fifth TMT joints with gross pain or arthritic changes and obvious structural deformity. Arthroplasty is an effective salvage operation mainly used in high-demand patients with severe TMT arthritis. Finally, we propose a recommended treatment algorithm (based on the literature and our experience), taking into account the specific indications for internal fixation, TMT arthrodesis and arthroplasty to optimize the individualized treatment.

***Data sources/Study selection*** Data from survey reports, descriptive, cross-sectional and longitudinal studies published from 2002 to 2012 on the topic of the injuries to the fourth and fifth tarsometatarsal joint on human and radiography studies were included.

***Data Extraction ***The data was extracted from online resources of American Orthopaedic Foot & Ankle Society, American Academy of Orthopaedic Surgeons, US National Library of Medicine, The MEDLINE.

***Conclusion*** It is important to comprehend the specific anatomical characteristics and grasp the strict indications, advantages and disadvantages of the ORIF, TMT arthrodesis and arthroplasty to optimize the individualized treatment of the fourth and fifth TMT joints injuries in a maximum extent.

## OVERVIEW

The fourth and fifth tarsometatarsal (TMT) joints, as a relatively independent unit of the lateral column in the foot, play an important role in a variety of activities of the foot. Various studies have indicated that the anatomic structures and function of the fourth and fifth TMT joints were different from the other three TMT joints.^1^^-^^3^ Injuries to the TMT joints are not common, with a reported incidence of one per 55000 yearly, accounting for 0.2% of all orthopedic injuries.^4^ Injuries to the fourth and fifth TMT joints can be seen in various traumatic events. However, current research focuses on the management of the medial three TMT joints with only scarce literature discussing the fourth and fifth TMT joints. At present, the surgical management of the fourth and fifth TMT injuries can mainly be classified as open reduction and internal fixation (ORIF), TMT arthrodesis and arthroplasty, each of which has specific indications, contraindications, advantages and disadvantages.^5^^-^^7^ Therefore, the surgical management should be individualized.

***The anatomical characteristics of the fourth and fifth TMT joints: ***The fourth and fifth TMT joints are designed to allow motion. Therefore, some special anatomical characteristics markedly influence the stability of the two joints. Structurally, the fourth and fifth TMT joints include the fourth and fifth cuboid-metatarsal (CMT) joints and the fourth-fifth intermetatarsal joint. Unlike the wedge-shaped configuration of the second cuneometatarsal joint, the fourth and fifth CMT joints are much flatter and even without significant convexity or concavity. Therefore, these two joints cannot benefit from the additional stability by a wedge-shaped structure.^8^ Cadaveric and imaging studies also revealed that the trapeziform or triangle articular contour of the fourth and fifth CMT joints would provide the lateral two joints a large range of motion (ROM).^1^^,^^9^ The fourth-fifth intermetatarsal joint connects the bases of the fourth and fifth metatarsals as a unit. However, due to the weaker attachment of the ligaments, the articulation is more loosened than the other intermetatarsal joints.

The stability of the fourth and fifth TMT joints largely depends on the ligamentous integrity. There is an independent joint capsule between the cuboid and the base of the fourth and fifth metatarsals. The dorsal and plantar ligaments are distributed on the surface of the capsule to enhance its stability. The fourth and fifth intermetatarsal interosseus ligament can also help to reinforce the stability.^10^^,^^11^ However, the ligaments do not contribute much to the stability of the fourth and fifth TMT joints.^12^ Additional supports to the fourth and fifth TMT joints are provided by the tendons. The interosseous muscles and plantar fascia can also maintain the stability by buffering the load from the dorsal to the plantar.^13^ The loosened articulation and limited strength of ligaments allow these two TMT joints a large ROM. We once measured the ROM of the lateral two TMT joints and found out that the fourth and fifth TMT joints had a maximal ROM of 18.5° and 20.2° respectively in sagittal plane.^3^ This large ROM provides the lateral foot great flexibility to keep the body balance.^14^ However, it is also a predisposing factor for the injuries to the fourth and fifth TMT joints.

**Fig.1:**
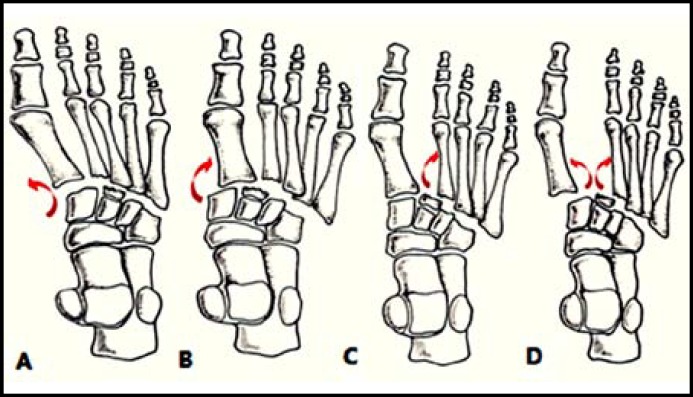
Patterns of injuries to the fourth and fifth TMT joints. **A, B:** In Myerson type A, all the TMT joints dislocated medially or laterally. **C:** In type B2, one or more of the lateral four TMT joints dislocated laterally. **D:** In type C2, the first TMT joint and the lateral four TMT joints dislocated in a divergent way.

***The injuries to the fourth and fifth TMT joints: ***The injuries to the fourth and fifth TMT joints usually result after trauma, which are easily misdiagnosed.^15^ The classification of the injuries to the fourth and fifth TMT joints is reflected in the modified Myerson classification system (Fig.1). Isolated injuries to the fourth and fifth TMT joints can also be seen in the literature, creating controversies as to the surgical management.^16^^,^^17^

Excessive forces are usually involved in direct or indirect injuries to the fourth and fifth TMT joints, producing a wide array of injury patterns from subluxation to fracture-dislocation. In direct injuries, the fourth and fifth metatarsals can undergo plantar or dorsal displacement depending on the direction of the force. Indirect injuries are most commonly associated with a longitudinal force applied to the forefoot in plantar flexion, which is then subjected to rotation and compression by abduction forces exerted onto the lateral foot.^18^ Two different plantar flexion mechanisms may lead to dorsal disruption of the fourth and fifth TMT joints. The first occurs in excessive plantar flexion of the ankle joint and dorsiflexion of the metatarsophalangeal joint with the TMT joint engaged along an elongated lever arm. Then the joint is “rolled over” by the imbalanced body and the load is transferred to the lateral foot. It commonly occurs when a person misses a step downstairs.^19^ Dorsal displacement can also occur when an axial load is applied to the heel in a fixed plantarflexed ankle with the toes in dorsiflexion, which is a common mechanism seen in athletes.^20^

The cuboid clamped between the calcaneous and fourth and fifth metatarsals is more sensitive to the indirect abduction forces to the forefoot, leading to the compressive or avulsion fracture. Therefore, the concomitant cuboid fracture in fourth and fifth TMT joints injuries may impact the options of the surgical method, since it is advised that shortening of the cuboid>3mm or the displacement of the CMT>1mm should be treated surgically.^21^

***The diagnosis of the fourth and fifth TMT joints injuries: ***It is not difficult to diagnose TMT injuries since the patients generally present with midfoot pain, swelling, bruising and difficulty in weight bearing. Careful examination must be carried out to rule out compression or laceration of the dorsalis pedis artery.^22^ Radiographically, severe malalignment between the fourth and fifth metatarsals and the cuboid can be seen in most cases. Frequently, disorders of other TMT joints or concomitant fractures of the navicular, cuneiforms and cuboid can simultaneously be seen. Fifth TMT joint injury should be distinguished from Jones fracture because the treatment of these two injuries is different.^23^

Subtle injuries to the fourth and fifth TMT joints are more difficult to diagnose. Careful palpation reveals acute tenderness along the involved TMT joints. Gentle passive supination or pronation of the forefoot with the hindfoot fixed usually elicits pain.^24^ Additional images such as weight bearing view, stress views, CT and MRI are always needed. If the medial border of the fourth metatarsal and cuboid is not continuous on the oblique view and the fourth and fifth metatarsals are more dorsal than the cuboid on the weight-bearing lateral view, disarticulation must be suspected.

Malunion of the fourth and fifth TMT joints can occur in misdiagnosed patients. The patients will complain of the abnormal foot posture with midfoot pain and difficulty in weight bearing or shoe wearing.^14^ The examiners can also find palpable bony prominences on the dorsum of the foot and the abnormal motion of the two joints. Malunited fracture sites and degenerative arthritis or traumatic arthritis changes to the fourth and fifth TMT joints can be revealed in radiographs. In some cases, pseudoarthrosis can also be found in a more pronated foot position.^25^

***The surgical management to the fourth and fifth TMT joints injuries: ***Injuries to the fourth and fifth TMT joints should be treated actively since long-term instability of the fourth and fifth TMT joints may encourage development of symptomatic midfoot arthritis. Initially, nonsurgical treatment can be used in patients with minor acute injuries or with mild TMT joint arthritis. However, if the symptoms fail to respond to nonsurgical treatment, surgery may be indicated.^14^

**Fig.2:**
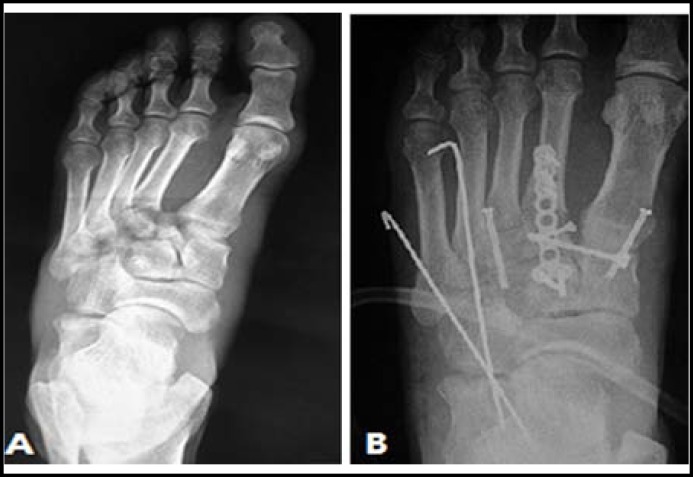
The use of K-wire to treat the injuries to the fourth and fifth TMT joints. **A: **Preoperative radiograph indicated Myerson type A injury with all the TMT joints dislocated laterally. **B:** Postoperative radiograph indicated the anatomic reduction with the fourth and fifth TMT joints being fixated with K-wires

***ORIF of the fourth and fifth TMT joints: ***ORIF is the first recommended option for the acute injuries to the fourth and fifth TMT joints from subluxation to fracture-dislocation. Kirschner-wire (K-wire) has been shown to be effective if anatomic reduction can be achieved and maintained.^5^ In open injuries of the fourth and fifth TMT joints, debridement with K-wire fixation and cast immobilization is regarded as the gold standard. K-wire fixation has the advantages of less damage to the articular surface and lower incidence of traumatic arthritis. The elastic fixation can help to keep the ROM of the fourth and fifth TMT joints, which will preserve the normal biomechanics of the midfoot as far as possible. We also measured the ROM after K-wire fixation and the maximal ROM of the fourth and fifth TMT joint would only decrease 4° and 5.4° in sagittal plane respectively.^3^ K-wires can also be used to treat the TMT joint complex injuries with other implants (Fig.2). Stavlas et al^26^ concluded that fixation of the three medial TMT joints with screws and of the lateral two TMT joints with K-wires could achieve good outcomes. However, K-wire fixation has no advantages in cases of severe comminution of articular surfaces and it can neither provide compression to the fracture site nor restore a damaged articular surface, which will easily lead to loosening or breakage of the K-wires and loss of reduction if the patient bears weight earlier than recommended.

Cortical screw fixation of the three medial TMT joints provides greater stability than K-wire fixation.^12^ But few authors suggest screw fixation for the fourth and fifth TMT joints, because this trans-articular fixation may aggravate the degeneration of the articular cartilage and accelerate the development of midfoot arthritis.^27^ But this controversial fixation method can be seen in some literatures. Saab^15^ reported to have fixated two cases of severe total TMT joints injuries with three cannulated screws and obtained the anatomic reduction. Sánchez-Gómez et al^28^ believed that the screws could treat the concomitant fracture of the cuboid or cuneiform simultaneously when the lateral TMT joints were fixated. The plate can also treat a concomitant fracture of the cuboid or the comminuted fractures of the fourth and fifth TMT joints. However, plate fixation is not traditionally recommended for larger dissection of the soft tissue and we believe that this rigid fixation is not biomechanically desirable at the lateral foot.^21^

***The arthrodesis of the fourth and fifth TMT joints: ***Whether arthrodesis can be used in the fourth and fifth TMT joints continues to be controversial. Most authors approve the motion-preserving procedures since they believed arthrodesis will increase the rate of nonunion and stress fracture.^29^^,^^30^ However, if gross pain, arthritic changes with obvious structural deformity or pseudoarthrosis is present in patients with malunion of the fourth and fifth TMT joints, arthrodesis may become an advisable option.^31^

Although there is no strict contraindication to the arthrodesis of the fourth and fifth TMT joints, the surgeons should know that an isolated lateral TMT arthrodesis can only be considered in cases of revision for implant failure or in symptomatic fourth and fifth TMT arthritis. Raikin et al^32^ once reported 23 cases of arthrodesis of the fourth and fifth TMT joints with severe deformity and pain. They determined the arthrodesis can produce good outcomes for pain relief and functional improvement, although 13 cases complained subjective stiffness of the lateral foot. However, compared to the pain relief, the patients were not concerned about the stiffness.

There was no correlation between the number of joints and the functional outcome in the patients with TMT joints arthrodesis.^33^ Pain will never be developed to the lateral TMT joints after isolated medial three TMT joints arthrodesis, and if no structural deformity or severe pain of the lateral TMT is present, the arthrodesis of the lateral TMT joints will not improve the overall curative effect either. However, as an independent unit of the foot, the fourth and fifth TMT joints can never be fused independently. As for the bone grafting for the arthrodesis site, it is also controversial. Although bone grafting is not always required and 100% union without bone grafting was also reported^34^, it is advised that bone grafting is best to be used, especially in the cases of wide joint resection. Park et al^16^ introduced a case of a single rectangular inlay bone grafting that traverses the fourth and fifth metatarsals and the cuboid. He believed this bone grafting could provide more graft to native bone contact than two dowel grafts traversing the fourth and fifth CMT joints individually.

***The arthroplasty of the fourth and fifth TMT joints: ***In high-demand patients with severe fourth and fifth TMT arthritis, the loss of mobility after arthrodesis may not be acceptable. Arthroplasty of the fourth and fifth TMT joints can be considered in such cases and it has been shown to be an effective salvage operation for the fourth and fifth TMT joints. At present, arthroplasty may be performed with tendon interposition or with a spherical ceramic interpositional implant.

The interpositional arthroplasty using soft tissue have been widely used in the treatment of the degenerative joint disease. In fourth and fifth TMT joints, the tissues used include either the peroneus tertius tendon or the peroneus brevis tendon. Mirmiran et al^35^ described the procedures to perform the tendon interpositional arthroplasty. An incision is made over the fourth-fifth CMT joints. The involved joints were exposed by subperiosteal dissection. The adjacent articular surfaces are removed by a sagittal saw. After the tendon of peroneus tertius or peroneus brevis is freed from its soft-tissue attachments and resected at its insertion site, it can be used as an interpositional graft. The tendon graft is then folded upon itself and maintained by a transfixing nonabsorbable suture. Finally, the tendon is placed into the articular spaces and secured by reapproximation of the capsular and periosteal tissues. He believed the procedure offered promise and a suitable alternative to arthrodesis since he reported three cases of fourth and fifth TMT arthritis with interposition arthroplasty. Follow-up evaluation demonstrated satisfactory pain relief without any complications. Berlet et al^6^ also performed the similar surgical procedures to the patients of traumatic lateral TMT joints arthritis. Seventy-five percent of the patients were satisfied with an average decrease in preoperative pain of 35%.

**Fig.3:**
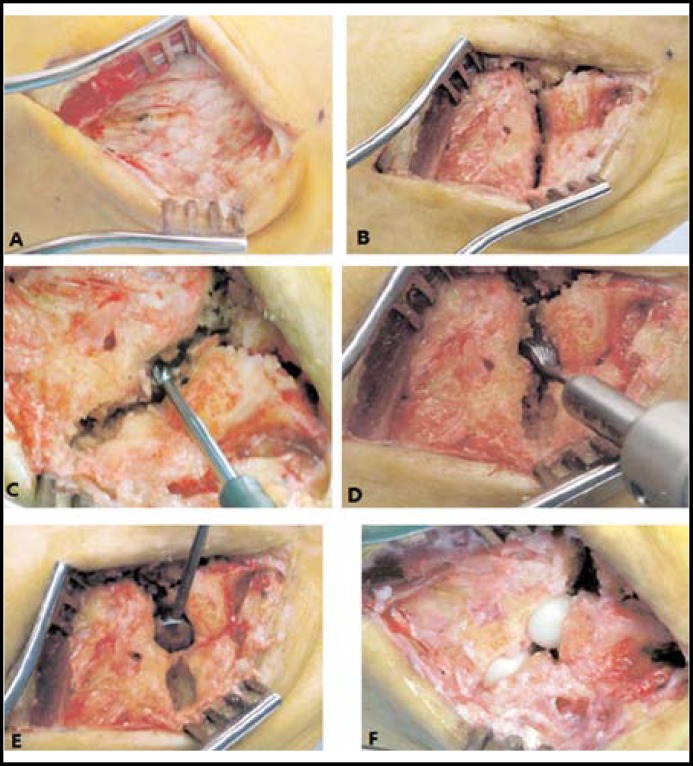
The interpositional arthroplasty with the Orthosphere® spherical ceramic implant.** A: **A dorsolateral approach over the lateral cuboid. **B:** Exposure of the lateral two TMT joints. **C:** Creation of a central hole in the opposing articular surfaces of each joint. **D:** Creation of semispherical recesses. **E:** The use of the sizing guides. **F:** Insertion of the spherical ceramic implant.

The use of the Orthosphere^®^ spherical ceramic (Wright Medical Technology, Arlington, TN, USA) allows for a technically simpler procedure and faster rehabilitation than the arthrodesis and it can also avoid the sacrifice of the tendons. The procedure is performed through a dorsolateral approach over the lateral aspect of the cuboid (Fig.3). Subperiosteal dissection gives access to the fourth and fifth TMT joints. The articular surfaces can be exposed after debridement of the osteophytes and synovium. Next, a central hole in the opposing articular surfaces of each joint is created using a starter burr. A finishing burr is then used to enlarge the recess on each side of the joint with the cortical rim and plantar ligaments being kept intact. Sizing guides are then used and implant sizes of 9 to 12mm are typically chosen. Mild joint distraction and free motion at the TMT joints are checked. Once the adequate size is confirmed, the implants can be placed into the TMT joints with plantar flexion applied to the metatarsals. Full ROM through the midfoot is then performed to confirm stability. Finally, the wound is closed and a splint is applied with the foot in a neutral position for two weeks. Shawen et al^29^ once treated 13 cases of the fourth and fifth TMT joints arthritis by ceramic interposition arthroplasty with the above procedures. The average postoperative AOFAS score had an 87% improvement over preoperative values and the visual analogue scale pain also improved 42%. Carpenter et al^36^ also reported a case of Orthosphere® zirconia ceramic implant for arthroplasty in fifth TMT joint arthritis. The patient finally could perform the daily activities without discomfort after six months. Complications such as dislocation have not been reported yet and the durability of the ceramic prosthesis is not known because of the few reported cases and short follow-up period. We believe that the long-term curative effect is yet to be determined.

## CONCLUSION

**Fig.4:**
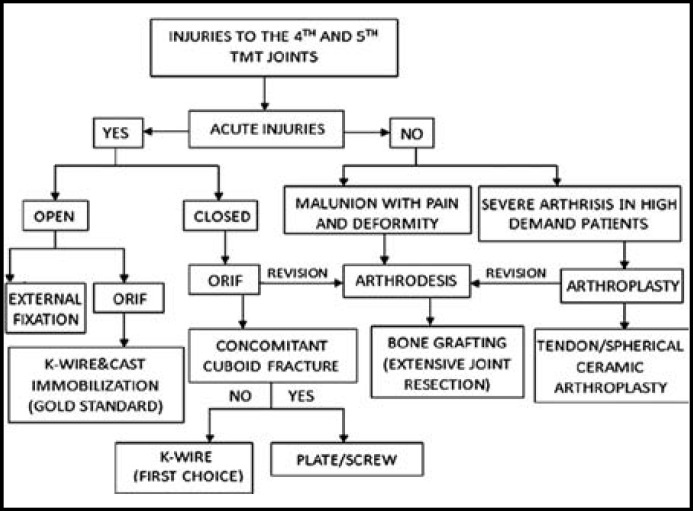
The surgical treatment algorithm for the injuries to the fourth and fifth TMT joints

The fourth and fifth TMT joints are designed different from other TMT joints. The specific anatomic structures allow the fourth and fifth TMT joints a large ROM. However, they are also predisposing factors to make the fourth and fifth TMT joints injured. The injuries to the fourth and fifth TMT joints should be treated individually. We conclude the following algorithm based on the literatures and our experience (Fig.4). ORIF is the first recommended option to the acute injuries to the fourth and fifth TMT joints and the K-wire is more widely used than other implants for the preservation of the ROM. However, the screw or the plate can be used to substitute the K-wire in the cases of concomitant fracture of cuboid. The arthrodesis of the fourth and fifth TMT joints is still controversial. It can be used in the cases of severe pain or severe arthritis changes of the lateral TMT joints with obvious structural deformity or pseudoarticulation formation. The complications of nounion and stress fracture should be noted and bone grafting is recommended in the cases of extensive joint resection. The arthroplasty is an effective salvage operation for the fourth and fifth TMT joints. It is mainly used in the severe arthritis of the TMT joints in high demand patients. The arthroplasty can be classified as tendon interpositional arthroplasty and spherical ceramic interpositional arthroplasty. The long-term curative effect and complications of the arthroplasty remains on observation.
